# Minimally Invasive and Open Gastrectomy for Gastric Cancer: A Systematic Review and Network Meta-Analysis of Randomized Clinical Trials

**DOI:** 10.1245/s10434-023-13654-6

**Published:** 2023-06-01

**Authors:** Matthew G. Davey, Hugo C. Temperley, Niall J. O’Sullivan, Vianka Marcelino, Odhrán K. Ryan, Éanna J. Ryan, Noel E. Donlon, Sean M. Johnston, William B. Robb

**Affiliations:** 1 Department of Surgery, Royal College of Surgeons in Ireland, Dublin 2, Republic of Ireland; 2 Department of Surgery, Trinity St. James’s Cancer Institute, Trinity, St. James’s Hospital, Trinity College Dublin, Dublin, Republic of Ireland; 3 Department of Surgery, Tallaght University Hospital, Dublin, Republic of Ireland; 4 Department of Surgery, St. Vincent’s University Hospital, Dublin, Republic of Ireland; 5 Department of Upper Gastrointestinal Surgery, Beaumont Hospital, Dublin 9, Co Dublin Republic of Ireland; 6 Department of Surgery, Midlands University Hospital, Tullamore, Co. Offaly Republic of Ireland

**Keywords:** Gastric cancer, Gastrectomy, Minimally invasive surgery, Surgical oncology, Oncological outcomes

## Abstract

**Background and Objectives:**

Optimal surgical management for gastric cancer remains controversial. We aimed to perform a network meta-analysis (NMA) of randomized clinical trials (RCTs) comparing outcomes after open gastrectomy (OG), laparoscopic-assisted gastrectomy (LAG), and robotic gastrectomy (RG) for gastric cancer.

**Methods:**

A systematic search of electronic databases was undertaken. An NMA was performed as per the Preferred Reporting Items for Systematic Reviews and Meta-Analyses (PRISMA)-NMA guidelines. Statistical analysis was performed using R and Shiny.

**Results:**

Twenty-two RCTs including 6890 patients were included. Overall, 49.6% of patients underwent LAG (3420/6890), 46.6% underwent OG (3212/6890), and 3.7% underwent RG (258/6890). At NMA, there was a no significant difference in recurrence rates following LAG (odds ratio [OR] 1.09, 95% confidence interval [CI] 0.77–1.49) compared with OG. Similarly, overall survival (OS) outcomes were identical following OG and LAG (OS: OG, 87.0% [1652/1898] vs. LAG: OG, 87.0% [1650/1896]), with no differences in OS in meta-analysis (OR 1.02, 95% CI 0.77–1.52). Importantly, patients undergoing LAG experienced reduced intraoperative blood loss, surgical incisions, distance from proximal margins, postoperative hospital stays, and morbidity post-resection.

**Conclusions:**

LAG was associated with non-inferior oncological and surgical outcomes compared with OG. Surgical outcomes following LAG and RG superseded OG, with similar outcomes observed for both LAG and RG. Given these findings, minimally invasive approaches should be considered for the resection of local gastric cancer, once surgeon and institutional expertise allows.

**Supplementary Information:**

The online version contains supplementary material available at 10.1245/s10434-023-13654-6.

Gastric cancer is the fifth most common cancer worldwide and third leading cause of cancer-related mortality.^1^ Its management paradigm has evolved with the advent of multimodal therapeutic strategies, including novel combinations of chemotherapeutic agents,^2^ radiotherapies,^3^ and immunomodulatory drugs,^4^ all of which may be tailored in accordance with patient and tumour factors to improve oncologic outcomes. This personalized approach is conducted with the intention of minimizing treatment-related toxicities, while simultaneously achieving the best pathological responses to conventional therapeutic strategies.^5,6^ Despite these advances, high-quality radical *en bloc* surgical resection of the tumour remains the cornerstone of management,^7,8^ Traditionally, gastrectomy was performed as an open procedure (OG), although more recently, minimally invasive surgical (MIS) approaches have undergone widespread adoption, including laparoscopic-assisted gastrectomy (LAG) and robotic-assisted gastrectomy (RG).^9,10^ Advocates of MIS hold this approach to be advantageous for several reasons, particularly reduced morbidity, and enhanced recovery and cosmesis.^11,12^ Nevertheless, OG remains the approach of choice for certain cases, as surgical approach is dependent on an array of patient, pathological, and societal parameters, as well as surgeon and institutional expertise.^13^

The first LAG was performed for gastric cancer in 1994,^14^ and this approach has subsequently been developed and refined.^15^ Several studies, including multicentre, prospective, randomized clinical trials (RCTs), have illustrated the non-inferiority of long-term oncological and survival outcomes following LAG and OG. Consequently, LAG is a well-established surgical approach in the management of early gastric carcinoma.^16–19^ Increasingly, LAG is currently emerging as feasible, safe and effective for radical resection of locally advanced distal gastric cancer.^20,21^ Nevertheless, there remains debate surrounding the differences in postoperative and oncological outcomes following LAG, as well as the considerable technical aspects and learning curve associated with the laparoscopic approach.^22–26^

Robot-assisted surgery has been proposed as a potential platform to overcome some of the limitations of conventional laparoscopy and has recently undergone widespread adoption by many specialists and surgical oncology units for the management of many solid organ cancers. This is principally due to the reported advantages of three-dimensional vision, enhanced skill acquisition (due to a shorter learning curve relative to laparoscopic surgery), increased operator dexterity, improved mobility in narrow areas that have restricted access, and improved ergonomics for the operating surgeon.^27,28^ However, while the first RG was performed in Japan 20 years ago by Hashizume et al.,^29^ the uptake of robotic surgery in upper gastrointestinal surgery has lagged considerably behind other surgical specialties.^30^ At present, data available in relation to robotic surgery is primarily obtained from studies of a retrospective design, typically involving outcomes regarding single-centre or single-surgeon experiences of using robotics for gastric cancer resections, with limited long-term oncological outcomes being reported.^7^ Therefore, there is a paucity of high-quality data evaluating the role of RG for resection of gastric tumours.^31–33^

Several RCTs and standard pairwise meta-analyses^32,34–37^ have attempted to determine the optimal surgical approaches used for the resection of gastric carcinoma, however consensus in relation to the oncological and surgical safety of LAG and RG relative to OG is yet to be determined. Importantly, two recent RCTs have reported short-term postoperative and survival outcomes following RG,^38,39^ and anticipation among gastroesophageal surgeons is that such minimally invasive approaches should enhance patient outcomes.^40^ Therefore, application of the network meta-analysis (NMA) methodology here is timely to allow simultaneous comparison (direct and indirect) of minimally invasive approaches to gastrectomy with OG using RCT data only.^41,42^

## Methods

A systematic review was performed in accordance with the Preferred Reporting Items for Systematic Reviews and Meta-Analyses (PRISMA) extension statement for reporting of systematic reviews incorporating NMAs of healthcare interventions.^43^ This study was registered with the International Prospective Register of Systematic Reviews (PROSPERO, CRD42022330440).

### Search Strategy

A formal systematic search of four electronic databases was performed in March 2022 for relevant titles. Details in relation to the search strategy can be found in Appendix 1 in the electronic supplementary material (ESM). Study-specific definitions and the research question determined using the Population, Intervention, Comparison, Outcomes (PICO) framework^44^ are outlined in detail in ESM Appendices 2 and 3.

### Eligibility Criteria

All published RCTs with full-text manuscripts comparing the outcomes of two or more methods of surgical intervention for gastric cancer (i.e., OG, LAG or RG) were included. The inclusion criteria for studies were (1) compared postoperative surgical outcomes (e.g., complications, estimated blood loss, lymph node yield [LNY], etc.) or long-term oncological and survival outcomes (e.g., disease recurrence, overall survival [OS], etc.); (2) were of a prospective, randomized design; (3) recruited patients aged 18 years or older undergoing surgery for known primary gastric cancer; and (4) studies had to have full-text manuscripts available. Note that when overlapping trial data were reported from two different sources, the source with the longest patient follow-up or largest sample size was included.

The exclusion criteria were (1) studies failing to fulfil the above inclusion criteria; (2) studies that only have results published in abstract form or from conference proceedings; (3) studies not published in the English language; or (4) studies in which the primary indication for the gastrectomy was not for gastric cancer (e.g., bariatric sleeve gastrectomy, etc.).

### Statistical Analysis

Descriptive statistics were used to outline the characteristics of the included trials. Data pertaining to recurrence, OS, morbidity, complications and readmission were expressed as dichotomous or binary outcomes, reported as odds ratios (ORs) with 95% confidence intervals (CIs). ORs were calculated using crude event RCT data, to compare interventions using per-protocol data, where applicable. Continuous data were calculated using mean values, standard deviations (SDs) and pooled mean variance, with differences expressed as weighted mean differences (WMDs). OG was the principal comparator for all analyses. Bayesian NMAs were conducted using netameta^45^ and Shiny packages^46^ for R. Point estimates of effect sizes were described with a 95% CI. Results were considered statistically significant at the *p* < 0.050 level if the 95% CI did not include a value of 1. Estimates of mean and SDs were calculated using standard statistical methods, where applicable.^47,48^ Rank probabilities were plotted against the possible ranks for all competing treatments. The confidence in estimates of the outcome was assessed using the Confidence in Network Meta-Analysis (CINeMA) tool.^49^

### Risk-of-Bias Assessment

Assessment of potential biases within the included RCTs was assessed using the Cochrane Collaboration tool (for RCTs).^50^ This assessment tool grades the risk of bias in each study as being high risk (marked in red), low risk (marked in green), or uncertain risk (marked in yellow) of bias across six categories. The critical appraisal was independently completed by two reviewers (HCT and MGD), and in the case of discrepancies in opinion, a third reviewer (NED) was asked to arbitrate.

## Results

### Literature Search

**Fig. 1:**
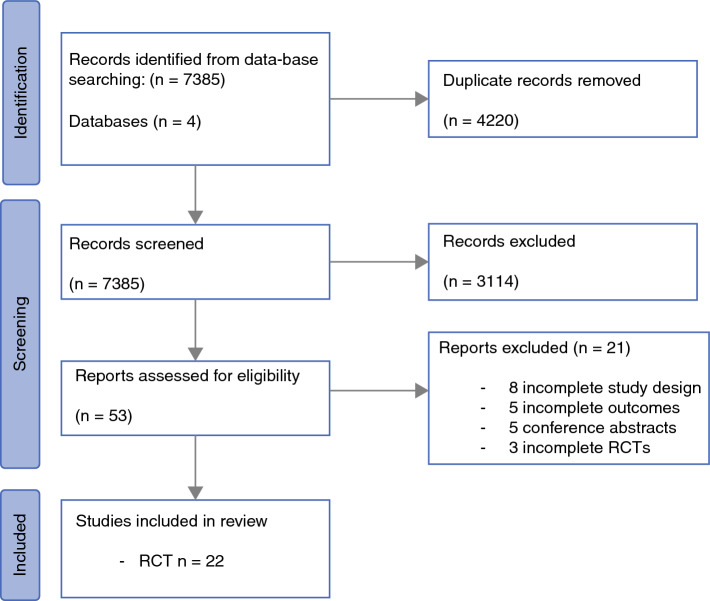
PRISMA flowchart outlining the systematic search process. *PRISMA* Preferred Reporting Items for Systematic Reviews and Meta-Analyses, *RCTs* randomized controlled trials

In total, 7385 articles were identified and 4220 duplicate articles were excluded. Thereafter, study titles and abstracts were screened, resulting in 53 studies being eligible for full-text review. Of these, 22 RCTs met the eligibility criteria and were included.^22–26,39,51–66^ The PRISMA flow chart is illustrated in Fig. 1.

### Study Characteristics

Overall, 6890 patients with gastric cancer were included, of whom 3212 patients underwent OG (46.6%, 3212/6890), 3420 patients underwent LAG (49.6%, 3420/6890) and 258 patients underwent RG (3.7%, 258/6890). The mean age at surgery was 60.9 years (range 54.5–72.0 years). Overall, 15/22 studies included distal gastrectomy (DG) only (68.2%), with 6/22 (27.3%) and 4/22 (18.2%) also including total gastrectomy (TG) and partial gastrectomy (PG), respectively. Overall, 40.9% reported outcomes for early gastric cancer (9/22), 18.2% for locally advanced gastric cancer (4/22), 9.1% for advanced gastric cancer (2/22), and 31.8% for resectable gastric cancer (7/22). The characteristics of the trials included in this meta-analysis are shown in Table 1. All ranking tables illustrating the data are presented in Table 2.

**Table 1:** Characteristics of trials included in the meta-analysis

Author, year	Country	Study period	Multicentre	Surgery	No. of patients	Age	M/F	Gastrectomy type	Cancer stage	Tumour size	Staging
Kitano^14^	Japan	1998–2001	No	OG LAG	14 14	60 ± 3 63 ± 3	8/6 9/5	DG	EGC	2.3 ± 0.3 2.3 ± 0.3	JCGC
Huscher^52^	Italy	1992–1996	No	OG LAG	29 30	63.6 ± 13 63.2 ± 12	21/8 18/12	DG	GC	NA NA	AJCC 5th
Lee^53^	Korea	2001–2003	No	OG LAG	23 24	59.5 ± 11 56.6 ± 11	15/8 11/13	DG	EGC	1.8 ± 1.6 1.4 ± 0.8	AJCC 5th
Hayashi^54^	Japan	1999–2001	No	OG LAG	14 14	62 ± 6.5 56 ± 5.7	13/1 9/7	DG	EGC	NA NA	NA
Cai^58^	China	2008–2009	Yes	OG LAG	47 49	60.2 ± 10.2 60.2 ± 9.8	37/10 39/10	PG, DG, TG	GC	4.3 ± 1.8 4.2 ± 2.0	JCGC 13th
Chen Hu^59^	China	2009–2011	No	OG LAG	20 22	64.5 ± 6.5 62.5 ± 6.7	12/8 10/12	DG	GC	NA NA	JCGC 13th
Takiguchi^55^	Japan	2003–2006	No	OG LAG	20 20	62.5 ± 3 61.5 ± 4.3	13/7 12/8	DG	EGC	2.4 ± 0.2 2.2 ± 0.5	NA
Kim^87^	Korea	2003–2005	No	OG LAG	82 82	54.5 ± 8.3 56.7 ± 7.5	47/35 47/35	DG	EGC	3.4 ± 2.1 3.4 ± 1.8	AJCC 6th
Yamashita^88^	Japan	2005–2008	No	OG LAG	32 31	61 ± 7.6 58 ± 9.6	25/7 17/14	DG	EGC	3.6 ± 2.4 3.9 ± 2.0	AJCC 5th
Cu^60^	China	2010–2012	No	OG LAG	142 128	57.5 ± 11.2 60.1 ± 12.6	98/44 88/40	PG, DG, TG	GC	NA NA	AJCC 7th
Aoyama^56^	Japan	2011	NA	OG LAG	13 13	63.8 ± 8.9 60.3 ± 11.8	7/6 7/6	DG	EGC	NA NA	JCGC 14th
Sh^57^	China	2010–2012	No	OG LAG	160 162	NA NA	NA NA	PG, DG, TG	AGC	NA NA	JCGC 13th
Kata^65^	Japan	2010–2013	Yes	OG LAG	459 462	64 ± 2.1 63 ± 1.8	275/184 275/184	DG, PG	ECG	2.5 ± 0.1 2.7 ± 0.3	JCGC 13th
Wang^26^	China	2014–2017	Yes	OG LAG	220 222	60.6 ± 10 59.4 ± 12	133/87 144/78	DG, TG	LAGC	3.9 ± 2.2 3.6 ± 1.8	AJCC 7th
Park^22^	Korea	2010–2011	Yes	OG LAG	96 100	60.1 ± 8.2 58.6 ± 8.9	65/31 69/31	DG	AGC	NA NA	AJCC 7th
Yu^20^	China	2012–2014	Yes	OG LAG	520 519	55.8 ± 11 56.5 ± 10	346/174 380/139	DG	LAGC	4.0 ± 2 4.0 ± 2	AJCC 7th
Kim^17^	Korea	2006–2010	Yes	OG LAG	611 644	57.8 ± 11 56.8 ± 11	412/200 425/219	DG	EGC	NA NA	AJCC 7th
Li^25^	China	2015–2017	No	OG LAG	50 45	61 ± 2.2 59 ± 3.2	34/16 32/13	DG	LAGC	2.5 ± 0.5 2.5 ± 0.4	AJCC 7th
Hyung^21^	Korea	2011–2015	Yes	OG LAG	498 513	59.6 ± 11 59.8 ± 11	346/152 370/143	DG	LAGC	NA NA	AJCC 7th
Vanderveen^89^	Netherlands	2015–2018	Yes	OG LAG	112 115	66.9 ± 12.1 67.9 ± 11.4	72/40 68/37	DG, TG	GC	NA NA	AJCC 8th
Jun Lu^39^	China	2017–2020	No	LAG RG	142 141	59.3 ± 11 59.4 ± 10	90/52 94/47	DG	GC	3.9 ± 1.9 3.5 ± 1.8	AJCC 8th
Ojima^66^	Japan	2018–2020	Yes	LADG RG	119 117	72 ± 8.3 71 ± 9.3	77/42 73/44	DG, TG	GC	3.2 ± 1.9 3.5 ± 2.4	AJCC 8th

*OG* open gastrectomy, *LAG* laparoscopic-assisted gastrectomy, *RG* robotic gastrectomy, *M* male, *F* female, *DG* distal gastrectomy, *PG* proximal gastrectomy, *TG* total gastrectomy, *NA* not available, *JCGC* Japanese Classification of Gastric Carcinoma, *AJCC* American Joint Committee on Cancer, *EGC* early gastric cancer, *GC* gastric cancer, *LAGC* locally advanced gastric cancer, *AGC* advanced gastric cancer

**Table 2:** SUCRA scores for the outcomes measures.

Parameter	OG	LAG	RG
Overall recurrence	0.732 (1st)	0.268 (2nd)	NR
Overall survival	0.563 (1st)	0.437 (2nd)	NR
Operative time	0.999 (1st)	0.001 (2nd)	0.000 (3rd)
Intraoperative blood loss	0.000 (3rd)	0.420 (2nd)	0.580 (1st)
Lymph node harvest	0.001 (3rd)	0.391 (2nd)	0.608 (1st)
Distance from proximal margin	0.006 (2nd)	0.996 (1st)	NR
Distance from distal margin	0.050 (2nd)	0.950 (1st)	NR
Length of incision	0.001 (2nd)	0.999 (1st)	NR
Wound complications	0.047 (2nd)	0.634 (1st)	0.319
Anastomotic leak	0.281 (2nd)	0.073 (3rd)	0.646 (1st)
Length of stay	0.000 (3rd)	0.274 (2nd)	0.725 (1st)
Days until sips	0.012 (3rd)	0.220 (2nd)	0.768 (1st)
Days until solids	0.099 (3rd)	0.679 (1st)	0.222 (2nd)
Days until flatus passed	0.000 (3rd)	0.257 (2nd)	0.743 (1st)
Days until ambulation	0.136 (3rd)	0.217 (2nd)	0.647 (1st)
Cost	0.646 (1st)	0.331 (2nd)	0.023 (3rd)
Tumour size	0.034 (3rd)	0.296 (2nd)	0.670 (1st)

*OG* open gastrectomy, *LAG* laparoscopic-assisted gastrectomy, *RG* robotic gastrectomy, *NR* not reported

### Primary Outcome Measures

#### Disease Recurrence

**Fig. 2:**
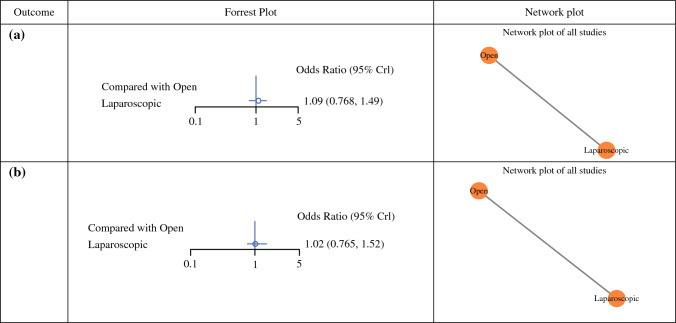
Forest and network plots with respect to (**a**) disease recurrence and (**b**) overall survival

Overall, 27.3% of studies reported outcomes with respect to disease recurrence (6/22). The mean follow-up was 56.4 months (range 22.1–99.8 months), and the overall recurrence rate was 9.1% (435/4775). LAG had the highest recurrence rate (9.5%, 226/2,373), followed by OG (8.7%, 209/2402). Of note, recurrence was not reported in any of the studies reporting outcomes following RG. When compared with an OG, the risk of disease recurrence was similar for those who underwent LAG (OR 1.09, 95% CI 0.77–1.49) (Fig. 2a) and ESM Appendix 2a].

#### Overall Survival

Overall, 31.8% of included studies reported outcomes for analysis in relation to OS (7/22). The mean follow-up was 56.4 months (range 22.1–99.8 months). For the overall patient cohort, 87.0% of patients were alive at follow-up (3302/3794). Of note, OS rates were identical for both OG and LAG (OS: OG, 87.0% [1652/1898] vs. LAG: OG, 87.0% [1650/1896]). OS was not reported in the RG groups. When compared with OG, OS was similar for those who underwent LAG (OR 1.02, 95% CI 0.765–1.52) (Fig. 2b and ESM Appendix 2b].

### Secondary Outcome Measures

#### Intraoperative Outcomes

**Fig. 3:**
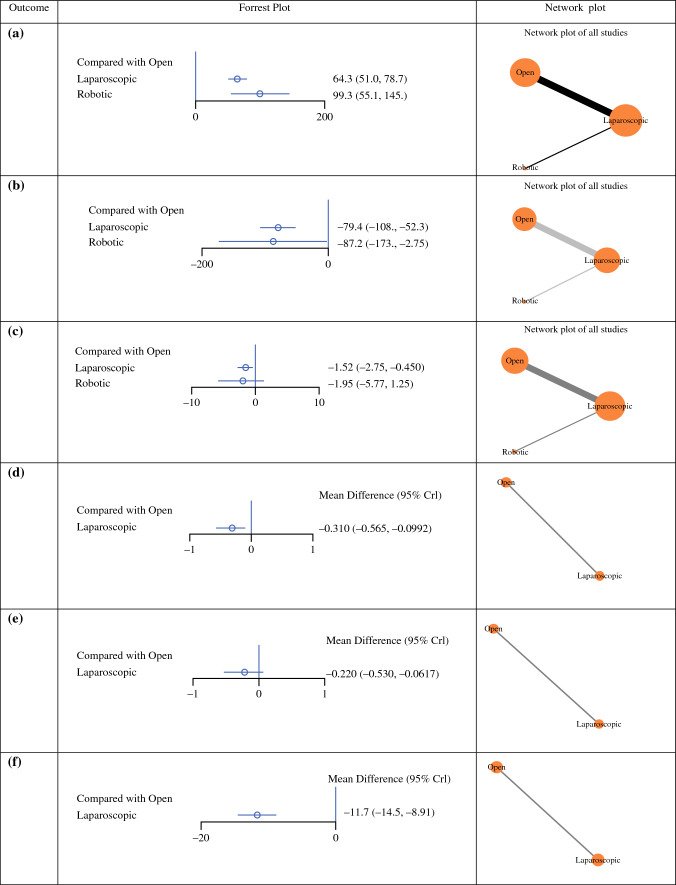
Forest and network plots with respect to intraoperative data. (**a**) Intraoperative time; (**b**) intraoperative blood loss; (**c**) lymph node harvest; (**d**) distance from the proximal margin; (**e**) distance from the distal margin; and (**f**) length of incision

All 22 RCTs reported outcomes on intraoperative time. LAG (OR 64.3, 95% CI 51.0–78.7) and RG (OR 99.3, 95% CI 55.1–145.0) were associated with significantly longer intraoperative duration than OG. Compared with LAG, intraoperative time was not significantly different to those who underwent RG (OR 34.96, 95% CI − 7.53 to 77.84) (Fig. 3a and ESM Appendix 3a). Of note, the rank probability was highest in the OG group (0.999), indicating the lowest intraoperative time associated with OG (Table 2).

In total, 95.5% (21/22) of the included studies reported outcomes in relation to intraoperative blood loss (IBL). Compared with OG, there was a significant reduction in IBL for those who underwent LAG (OR − 79.4, 95% CI − 108.0 to − 52.3) and the robotic group (OR − 87.2, 95% CI − 173.0 to − 2.75) (Fig. 3b and ESM Appendix 3b). Furthermore, there was a non-significant reduction in IBL in those undergoing RG compared with those undergoing LAG (OR − 7.83, 95% CI − 88.83 to 73.02).

Overall, 50.0% of included studies reported on the number of LNs harvested (11/22). Compared with OG, LAG showed a significantly decreased LN yield (OR − 1.52, 95% CI − 2.75 to − 0.45), whereas there was a non-significant difference in LNs harvested for those who underwent RG (OR − 1.95, 95% CI − 5.77 to 1.25) (Fig. 3c and ESM Appendix 3c)*.*

In total, 31.8% of the included studies reported on the distance from the proximal margin (7/22). This distance from the proximal margin was significantly lower in those undergoing LAG compared with OG (OR − 0.310, 95% CI − 0.565 to − 0.0992) (Fig. 3d and ESM Appendix 3d). The rank probability was highest in those undergoing LAG (0.99), indicating a decreased distance from the proximal margin in those undergoing LAG (Table 2).

In total, 27.3% of the included studies reported on the distance from the distal margin (6/22). The distance from distal margin was similar to those who underwent LAG and OG (OR − 0.220, 95% CI − 0.530 to 0.0617) (Fig. 3e and ESM Appendix 3e). The rank probability was highest in those undergoing LAG (0.95), indicating a decreased distance from the distal margin associated with LAG (Table 2).

In total, 36.4% of the included studies reported on the length of surgical incision (8/22). This was significantly shorter in those undergoing LAG compared with OG (OR − 11.7, 95% CI − 14.5 to − 8.91) (Fig. 3f and ESM Appendix 3f). The rank probability was highest in those undergoing LAG (0.99), indicating a decreased length of surgical incision associated with LAG (Table 2).

#### Postoperative Outcomes

Overall, 95.5% of included studies reported outcomes in relation to overall morbidity (21/22). Compared with OG, there was a significant reduction in morbidity in those undergoing LAG (OR 0.80, 95% CI 0.67–0.95) and RG (OR 0.35, 95% CI 0.19–0.62), respectively. Compared with LAG, those undergoing RG had a significant reduction in overall morbidity (OR 0.43, 95% CI 0.25–0.76) [ESM Appendices 4 and 5].

In total, 59.1% of included studies reported outcomes in relation to major morbidity (13/22). Compared with OG, there was similar major morbidity in those undergoing LAG (OR 1.15, 95% CI 0.79–1.65) and RG (OR 0.43, 95% CI 0.16–1.17). Compared with LAG, there was a significant reduction in major morbidity in those undergoing RG (OR 0.38, 95% CI 0.14–0.99) [ESM Appendices 4 and 5].

In total, 68.2% of included studies reported outcomes in relation to perioperative mortality (15/22). Compared with OG, there was similar perioperative mortality in those undergoing LAG (OR 0.90, 95% CI 0.43–1.89) and RG (OR 0.91, 95% CI 0.05–16.18). Compared with LAG, perioperative morality was similar for those who underwent RG (OR 0.99, 95% CI 0.06–15.88) [ESM Appendices 4 and 5].

#### Complications

Overall, 50.0% of RCTs reported on wound complications (11/22). Compared with OG, there were similar wound complications for undergoing LAG (OR 0.628, 95% CI 0.30–1.18) and RG (OR 1.03, 95% CI 0.11–10.70). Compared with LAG, wound complications were similar for those who underwent RG (OR 1.65, 95% CI 0.20–15.91) [ESM Appendices 4 and 5].

In total, 40.9% of included studies reported on cardiac complications (9/22). Compared with OG, cardiac complications were similar for those who underwent LAG (OR 1.47, 95% CI 0.68–3.17) and RG (OR 8.1, 95% CI 0.96–68.2). Compared with LAG, cardiac complications were similar for those who underwent RG (OR 0.18 95% CI 0.02–1.29). Of note, however, one of the two RCTs evaluating RG illustrated a significant increase in cardiac complications (OR 33.5, 95% CI 1.97–568.6) [ESM Appendices 4 and 5].

In total, 81.8% of included studies reported on respiratory complications (18/22). Compared with OG, there was a similar rate of respiratory complications for those who underwent LAG (OR 0.85, 95% CI 0.62–1.17) and RG (OR 0.65, 95% CI 0.29–1.46). Compared with LAG, the risk of respiratory complications was similar for those who underwent RG (OR 0.76, 95% CI 0.36–1.61) [ESM Appendices 4 and 5].

In total, 59.1% of included studies reported on pancreatic complications (13/22). Compared with OG, the risk of pancreatic complications was similar for those who underwent LAG (OR 0.72, 95% CI 0.34–1.51) and RG (OR 0.20, 95% CI 0.01–4.32). Compared with LAG, the risk of pancreatic complications was similar for those who underwent RG (OR 0.14, 95% CI 0.01–2.82) [ESM Appendices 4 and 5].

In total, 18.2% of included studies reported on VTE (4/22). Compared with OG, the risk of VTE was similar for those who underwent LAG (OR 0.90, 95% CI 0.43–1.89) and RG (OR 0.91, 95% CI 0.05–16.18). Similarly, the risk of VTE was comparable between LAG and RG (OR 0.99, 95% CI 0.06–15.88) [ESM Appendices 4 and 5].

Overall, 72.7% of included studies reported on anastomotic leak (16/22). Compared with OG, the risk of anastomotic leak was similar for those who underwent LAG (OR 1.17, 95% CI 0.69–1.96) and RG (OR 0.70, 95% CI 0.12–3.67). Compared with LAG, the risk of anastomotic leak was similar for those who underwent RG (OR 0.61, 95% CI 0.11–2.80) [ESM Appendices 4 and 5].

In total, 22.7% of included studies reported on anastomotic stenosis (5/22). Compared with OG, the risk of anastomotic stenosis was similar for those who underwent LAG (OR 0.84, 95% CI 0.19–3.71) and RG (OR 0.14, 95% CI 0.00–4.03). Compared with LAG, the risk of anastomotic stenosis was similar for those who underwent RG (OR 0.17, 95% CI 0.01–3.39) [ESM Appendices 4 and 5].

#### Recovery

Overall, 45.5% of included studies reported outcomes on length of hospital stay, in days (10/22). When compared with an OG, there was a significant reduction in length of hospital stay for those who underwent LAG (OR − 1.18, 95% CI − 2.01 to − 0.48). Compared with OG, length of hospital stay was similar for patients who underwent RG (OR − 1.78, 95% CI − 4.15 to 0.419). Compared with LAG, length of hospital stay was similar for patients who underwent RG (OR 0.60, 95% CI − 1.54 to 2.78) [ESM Appendices 6 and 7].

In total, 40.9% of included studies reported on the number of days until a patient could ingest sips of fluids (9/22). Compared with an OG, there was a non-significant reduction in the number of days until a patient could ingest sips of fluids for those undergoing LAG (OR − 0.416, 95% CI − 0.826 to 0.0227) and RG (OR − 0.679, 95% CI − 1.53 to 0.245). Compared with LAG, the number of days until a patient could ingest sips of fluids was comparable for those who underwent RG (OR − 0.26, 95% CI − 1.02 to 0.54) [ESM Appendices 6 and 7].

In total, 31.8% of included studies reported on the number of days until a patient could ingest solid food (7/22). Compared with OG, the number of days until a patient could ingest solid food was similar for those who underwent LAG (OR − 0.620, 95% CI − 1.81 to 0.55) and RG (OR 0.379, 95% CI − 2.79 to 3.54). Compared with LAG, the number of days until a patient could ingest solid food was similar for those who underwent RG (OR 1.00, 95% CI − 1.95 to 3.95) [ESM Appendices 6 and 7].

In total, 77.3% of included studies reported on the number of days until a patient could first pass flatus (17/22). Compared with OG, there was a significant reduction in the number of days until a patient could first pass flatus for those undergoing both LAG (OR −  0.455, 95% CI −  0.650 to −  0.259) and RG (OR − 0.61, 95% CI − 1.13 to − 0.080). Compared with LAG, there was a non-significant reduction in the number of days until a patient could first pass flatus for those undergoing RG (OR − 0.15, 95% CI − 0.64 to 0.34) [ESM Appendices 6 and 7].

In total, 36.4% of included studies reported on days till first ambulation (8/22). Compared with OG, there was a similar number of days to first ambulation for those who underwent LAG (OR − 0.15, 95% CI − 0.81 to 0.50) and RG (OR − 0.40, 95% CI − 1.69 to 0.89). Compared with LAG, days to ambulation were similar for those who underwent RG (OR − 0.25, 95% CI − 1.36 to 0.87) [ESM Appendices 6 and 7].

Overall, 18.2% of included studies reported on readmission (4/22). Compared with OG, the rates of readmission were similar to those who underwent LAG (OR 0.91, 95% CI 0.53−1.56) or RG (OR 0.92, 95% CI 0.12–7.10) [ESM Appendices 6 and 7]. Compared with LAG, the rate of readmission was similar for those who underwent RG (OR 0.99, 95% CI 0.14–7.15).

#### Tumour Size

Overall, 68.2% of included studies reported tumour size (15/22). Compared with OG, tumour size was similar to those who underwent LAG (OR − 0.13, 95% CI − 0.32 to 0.06) and RG (OR − 0.24, 95% CI − 0.75 to 0.30). Compared with LAG, tumour size was similar to those who underwent RG (OR − 0.11, 95% CI − 0.59 to 0.40) [ESM Appendices 8 and 9].

#### Cost Effectiveness

Overall, 13.6% of included studies reported on cost (3/22). RG was significantly more expensive than LAG (OR 3258.00, 95% CI 3204–59,3311.41) [ESM Appendices 8 and 9].

#### Risk of Bias

All 22 of the included studies were ‘low’ risk of bias for most categories, using the Cochrane Collaboration risk-of-bias assessment for RCTs. In brief, three of the included RCTs had ‘low’ risk of bias, 12 RCTs had ‘some’ risk of bias, and 7 RCTs illustrated evidence of ‘high’ risk of bias. Comprehensive summaries of the risk-of-bias assessment are outlined in ESM Appendix 10.

## Discussion

The management paradigm for gastric carcinoma has evolved such that multimodal therapeutic strategies are now pragmatically tailored to each patient, which has translated into enhanced oncological and survival outcomes.^67^ In addition, the inclusion of quality-of-life (QoL) outcome measures is now routinely utilized to establish the impact of both local and systemic therapies on host (or biological) function, to determine whether the oncological benefit of such treatments offset their associated toxicities and morbidity.^68^ This NMA was performed to establish the oncological and surgical safety of MIS gastrectomy compared with conventional OG, including data from 6890 patients treated for primary gastric carcinoma in 22 independent RCTs. This analysis illustrated the non-inferiority of LAG compared with OG with respect to survival, while highlighting the improved surgical and recovery outcomes associated with the MIS approaches, supporting their use where possible. Consequently, LAG should be considered for patients with primary resectable gastric cancer, providing that surgeon and institutional expertise allows, echoing the previous comprehensive results of a standard pairwise meta-analysis of RCTs performed by Lou et al. in 2022,^69^ albeit limited by the inclusion of studies performed three decades previously.

Traditionally, extensive locoregional resection using OG was the standard of care for resection of gastric carcinoma, which has since been surpassed by the adoption of both laparoscopic and robotic approaches as routine.^70,71^ Importantly, this study illustrated the non-inferiority of LAG relative to OG with respect to long-term oncological and survival outcomes, despite reduced nodal yields harvested and closer distances to specimen margins with LAG. OS was identical for both OG and LAG (both 87.0%) at approximately 5 years’ follow-up, with similar recurrence observed for both (9.5% vs. 8.7%), which likely represents a significant proportion of patients with early-stage disease, limiting the translatability of these results into the locally advanced setting. However, notably, disease recurrence and OS outcomes are as yet unavailable for patients undergoing RG compared with the other modalities. Therefore, while this study comprehensively establishes the non-inferiority of LAG versus OG for disease recurrence and OS, the absence of RCT data for RG limits the synthesis of any similar level 1 conclusions regarding the oncologic efficacy of RG. In a previous meta-analysis of 19 non-randomized, observational studies including 7275 patients, Ma et al. reported similar OS (hazard ratio [HR] 0.95, 95% CI 0.76–1.18), recurrence-free survival (HR 0.91, 95% CI 0.69–1.21) and disease recurrence (HR 0.90, 95% CI 0.67–1.21) for patients undergoing RG versus LAG.^72^ Furthermore, a previous propensity-matched analysis performed by Obama et al. demonstrated the non-inferiority of RG relative to LAG for disease recurrence (6.7% vs. 5.0%) and OS (8.9% vs. 11.6%).^73^ Interestingly, however, there was an increase in locoregional recurrence rates following RG relative to LAG (42.9% vs. 30.8%). In the absence of RCT data, the results from studies such as that by Obama et al. are important to highlight the potential risks and fundamental challenges to introducing and implementing new surgical techniques, albeit being limited due to its single-centre, retrospective design. However, it is important that further rigorous scientific evaluation of RG with prospective, randomized studies are conducted to ensure patient safety and to avoid the unexpected issues that have arose during the early adoption of other new surgical techniques for cancer,^74,75^ while remaining cognizant of the fact that premature adoption, inadequate proctoring and suboptimal execution, rather than any issue with the technique itself, may be to blame.^76^

While the survival outcomes for LAG and OG are equivocal, it is imperative that the other results in this NMA are considered when selecting the optimal technique for performing gastrectomy for gastric cancer, particularly in terms of the enhanced patient recovery and reduced complication rate associated with the MIS approaches, which coincide with the robust implementation of enhanced recovery after surgery (ERAS) protocols in contemporary surgical oncology.^77^ Patients undergoing LAG experienced a reduction in IBL, shorter surgical incisions, reduced distance from proximal margins, shorter postoperative hospital stays, and, most importantly, reduced morbidity post-resection. When interpretating these data, these important findings tip this study in favour of minimally invasive techniques, particularly when these data support the comparability of LAG and OG regarding long-term follow-up. MIS is advantageous as patients tend to be subject to less physiological stress, immunologic burden, faster recovery times, lower complication rates, and less immediate and long-term burden on healthcare resources.^78^ Therefore, MIS techniques may prove advantageous in improving cost effectiveness in the long-term, despite the greater direct cost associated with such surgical approaches as described in the current analysis. Accordingly, this study further validates the current paradigm shift towards adopting MIS techniques where possible, as these approaches are associated with longer operative duration relative to OG, as well as RG being significantly more expensive than LAG in the current NMA, coupled with emerging evidence suggesting an increased risk of cardiovascular complications, rendering patient selection imperative if RG is being contemplated during multidisciplinary discussion. Therefore, this study highlights the premise for LAG to be utilized for primary gastric resection where feasible, should institutional expertise allow, with further evaluation of RG approaches required to determine what benefit, if any, this approach may have over LAG.

Despite the absence of survival data, this study does provide preliminary data in support of RG, which was associated with reduced morbidity, major morbidity, and similar cardiorespiratory, pancreatic and other significant postoperative complications in this study compared with OG and LAG. Moreover, when compared with LAG, patients undergoing RG had a significant reduction in overall morbidity (OR 0.43, 95% CI 0.25–0.76), further potentiating RG as a pragmatic minimally invasive approach to gastrectomy in patients with early gastric cancer. These are important findings that strengthen the perceived benefit associated with RG, particularly when the application of robotic technology is an attractive addition to the surgeons’ armamentarium, due to the theoretical advantages over conventional laparoscopy, including improved dexterity, enhanced visualization, and superior ergonomics.^79^ Shortcomings of robotic surgery include the longer operative time and increased expense associated with this approach, as well as purchasing and maintaining equipment and training operators, a steep learning curve, and poorer cost effectiveness in low-volume centres.^80–83^ While the data suggest reasonable equipoise between RG and LAG surgery in terms of morbidity and recovery,^72^ the argument that robotic instrumentation may allow for improved mobility in narrow areas with restricted access, for example, at the diaphragmatic hiatus and when performing anastomoses, seems plausible.^84^ Another example is lymphadenectomy, where the removal of the D2 nodes is considered the standard surgical procedure for the majority of patients with resectable gastric carcinoma.^85^ Controversary remains in relation to resection beyond D2 for cases of advanced disease, for several reasons, including the reduced operative freedom, the significant difficulty controlling haemorrhage, and the relative ease of trauma to local structures.^86^ The results of the present NMA show a significant reduction in LNY following LAG, with similar outcomes observed following OG and RG, indicating that RG may offer an advantage over LAG for technically difficult manoeuvres such as extensive lymphatic resection during ‘D2 plus’ lymphadenectomy for advanced gastric cancer.^85^

The current analysis is subject to limitations. First, as described in detail, none of the two included RCTs reported survival outcomes following RG, limiting the conclusions that may be drawn from the current study. Second, the studies included in this review failed to provide survivorship data that may inform the psychosocial impact of OG, LAG, and RG on patients’ QoL following resection. Third, there are several competing factors that may confound the data presented in this study; these include the prescription of (neo)adjuvant chemo-, radio-, or immunotherapeutic agents to improve survival outcomes, as well as the impact of surgical approach in the context of early, locally advanced, or advanced gastric carcinoma. Unfortunately, attempts to perform analyses allowing for correction of such factors have been futile, thus limiting these results. Finally, evaluation of surgeon-specific proficiency and the influence of the centralisation of gastric cancer treatment to high-volume centres was not evaluated to determine their impact on clinical outcomes. Therefore, those responsible for the provision of the next generation of prospective, randomized gastrectomy trials should consider these confounding factors at the time of trial design to ensure the optimisation of data outcomes.

## Conclusion

This analysis demonstrates the non-inferiority of oncological and surgical outcomes for OG and LAG in patients being treated predominantly for early gastric cancer following 5 years of follow-up. Moreover, surgical outcomes following minimally invasive gastrectomy superseded those following OG, with enhanced outcomes observed for both LAG and RG. This highlights the oncological and surgical safety of LAG relative to OG, while also illustrating that the short-term surgical, morbidity and recovery outcomes following RG are comparable with LAG. Given these findings, LAG may be considered for patients with primary resectable gastric cancer, providing that surgeon and institutional expertise allows, however further RCTs are warranted before definitive conclusions may be drawn in the setting of advanced disease. However, given the challenges in adopting high-quality LAG for all cases, evaluation of new techniques that might make the procedure easier and thereby reduce the incidence of poor oncological and functional outcome are warranted. The concept of utilizing RG in these circumstances offers a number of theoretical but as yet unproven advantages, apart from LNY as was demonstrated in this analysis. In time, as more institutions employ an RG approach, it is likely that surgical and institutional expertise will facilitate further evaluation of the MIS techniques to determine the relative advantages and disadvantages of both techniques, which have not been extensive explored in this study. In the interim, technique selection should be based on individual tumour characteristics and patient expectations, as well as surgeon and institutional expertise.

## Supplementary Information

Below is the link to the electronic supplementary material.

Supplementary file1 (DOCX 1611 kb)

## Data Availability

Data can be made available upon reasonable request from the corresponding author.
