# Supplementary material for: Minimally Invasive and Open Gastrectomy for Gastric Cancer: A Systematic Review and Network Meta-Analysis of Randomized Clinical Trials
Source: Ann Surg Oncol. 2023 Jun 1;30(9):5544–57. doi: 10.1245/s10434-023-13654-6 (PMC10409677; doi:10.1245/s10434-023-13654-6)
Supplement: Supplementary file 1 — Supplementary file1 (DOCX 1611 kb) [file 10434_2023_13654_MOESM1_ESM.docx]

**Minimally Invasive and Open Gastrectomy for Gastric Cancer – A Systematic Review and Network Meta-analysis of Randomized Clinical Trials**

**Supplementary Appendices**

**Supplementary Appendix 1: Search Strategy**

A formal systematic search of the PUBMED, SCOPUS, EMBASE and Cochrane Central Register of Controlled Trials (CENTRAL) electronic databases was performed for relevant titles. This search was performed in March 2022. This search was performed by two independent reviewers (HCT & MGD), using a predetermined search strategy that was designed by the senior authors. this electronic systematic research will be carried out using PubMed, Scopus, Embase database, and the Cochrane Central Register of Controlled Trials of articles identifying minimally Invasive and open Gastrectomy for Gastric Cancer surgery The following search terms were used: “robotic-assisted*”, “laparoscopic*”, "open*” “gastrectomy*”, “gastric cancer”. The symbol “*” was used to allow variations on a word stem to be included in the search results. Furthermore, the following MeSH (medical subject headings) were used: (robotic-assisted[MeSH] OR laparoscopic[MeSH] OR open [MeSH)) AND (gastrectomy[MeSH] OR gastric cancer[MeSH]).

Manual cross-referencing of reference lists from previous systematic reviews, meta-analyses, and included trials was undertaken to ensure all RCTs published with full-text manuscripts were captured in this study. Manual removal of duplicate studies was performed before all titles were screened, where studies considered to be appropriate had their abstracts and/or full text reviewed. Retrieved studies were reviewed to ensure inclusion criteria were met, with discordances in opinion resolved through consultation with a third author (NED). Data extraction was also performed by two independent reviewers (HCT & MGD), with study details, basic patient clinicopathological characteristics and surgical data all recorded. Furthermore, information extracted was based on the PICO framework (Population, Intervention, Comparator, Outcomes). The latest database search was performed in late March 2022.

In total, 7,385 articles were identified based on the literature search of the PUBMED, SCOPUS, EMBASE, and the Cochrane Central Register of Controlled Trials (CENTRAL) databases. Of those, 4,220 articles were duplicate articles which were excluded. Thereafter, study titles and abstracts were screened, before 3,114 articles were excluded. This left 53 studies which had their full-text manuscripts assessed for eligibility. Of these, 21 articles were excluded, leaving 22 RCTs which met the eligibility criteria and were included. The updates of these trials were also included for qualitative and quantitative analysis (1-7).

**Supplementary Appendix 2: Definitions**

Gastrectomy: the anatomic surgical resection of the stomach with or without associated regional lymphadenectomy (1).

LAG: minimally invasive laparoscopic technique for anatomic surgical resection of the stomach with or without associated regional lymphadenectomy, followed by mini-laparotomy (with incision less than 10cm in length) for gastrointestinal reconstruction (20).

RG: the use of the robotic technique a minimally invasive laparoscopic technique for anatomic surgical resection of the stomach with or without associated regional lymphadenectomy, (8).

Early gastric cancer: invasive gastric carcinoma with deepest local invasion of the submucosa (T1), with or without lymph node metastasis (Nx) (9).

Locally advanced gastric cancer: invasive gastric carcinoma with deepest local invasion of the muscularis propria (T2), with or without lymph node metastasis (Nx) (10).

Advanced gastric cancer : invasive gastric carcinoma with deepest local invasion of the serosa (T3-4b) or beyond, with or without lymph node metastasis (N0-3b). These definitions were performed in accordance with the American Joint Committee on Cancer Tumour Nodes Metastases (AJCC-TNM) staging (8^th^ edition) (1, 11).

**Supplementary Appendix 3: Population, Intervention, Comparison, Outcomes (PICO)**

Using the PICO framework (12) , the aspects the authors wished to address were:

Population – Patients aged 18 years or older undergoing gastrectomy surgery for known resectable gastric cancer,

Intervention – Any patient who was randomised to undergo OG for resection of their primary gastric cancer,

Comparison – Any patient who was randomised to undergo LAG or RG for resection of their primary gastric cancer

Outcomes - The primary outcomes of interest were as follows:

- Disease recurrence, which was defined as experiencing local, regional or distant disease recurrence of their primary gastric carcinoma,
- OS, which was defined as mortality due to any cause.

The secondary outcomes of interest were as follows:

- Intraoperative outcomes:

Operative time: the mean operation duration of the gastrectomy from knife to skin to completion of application of skin dressings (measured in minutes).

Intraoperative blood loss (IBL): the millilitres of blood lost by a patient during the resection of a primary gastric cancer.

LNY: the number of locoregional LNs resected during the resection of a primary gastric cancer.

Distance from the margins: the shortest distance of non-cancerous tumour (measured in centimetres (cm)) from tumour edge measured at distal and proximal ends of the tumour specimen.

Length of incision: the largest abdominal incision made during the gastrectomy in cm.

- Postoperative outcomes:

Overall morbidity: any documented complication in the post-operative setting, classified in accordance with the Clavien-Dindo classification for post-operative complications (13),

Major morbidity: any documented complication meeting Clavien-Dindo classification of 2 or greater in the post-operative setting following gastrectomy (13),

Wound complications: surgical site infection, wound dehiscence, and wound breakdown.

Cardiac complications: any complication of cardiac origin including cardiac arrest, myocardial infarction, cardiac arrythmias, and pulmonary oedema.

Respiratory complications: any complication of respiratory origin including pleural effusions, atelectasis, pneumonia, and pneumothoraces

Pancreatic complications: any complication of pancreatic origin including peripancreatic collections, fistulae, and pancreatitis.

Venous thromboembolism (VTE): any thrombus of the venous system which complicated the patients recovery following gastrectomy.

Anastomotic leak (AL): full thickness gastrointestinal defect involving gastric, anastomosis, or staple line, irrespective of presentation or method of identification (14).

Anastomotic stenosis: any form of narrowing in the anastomosis region by contrast swallow studies or gastroscopy (less than ≤10mm in diameter) and any symptom of dysphagia when swallowing solid, semi-solid or liquid nourishment, which then required, endoscopic dilation (15).

- Recovery measures

Length of stay: calculated as the number of days from admission until the day of discharge from the hospital following gastrectomy.

Days till sips, solids, flatus passed and ambulation: calculated as the number of days from the day of operation until the first day sips were ingested, solids were ingested, flatus was passed, and ambulation post-operatively.

Readmission: any subsequent admission to the hospital following discharge in the post-operative period due to a sequelae of the patient’s gastrectomy.

- Others

Tumour size: the maximal diameter in cm of the invasive component of the primary tumour as reported on pathologic examination.

Cost: the overall price of procedure, reported in US dollars.

**Supplementary Appendix 4: Intraoperative data**

Forest and network plots with respect to Postoperative data (A) overall morbidity, (B) major morbidity, (C) perioperative mortality, (D) wound complications, (E) cardiac complications, (F) respiratory complications, (G) pancreatic complications, (H) Venous thromboembolism, (I) Anastomotic leak and (J) Anastomotic stenosis

| **Outcome** | **Forrest Plot** | **Network plot** |
| --- | --- | --- |
| **A** | **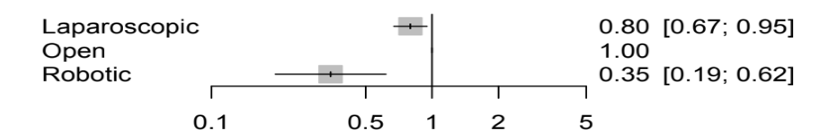** | 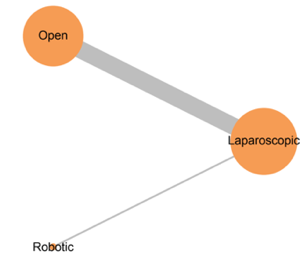 |
| **B** | **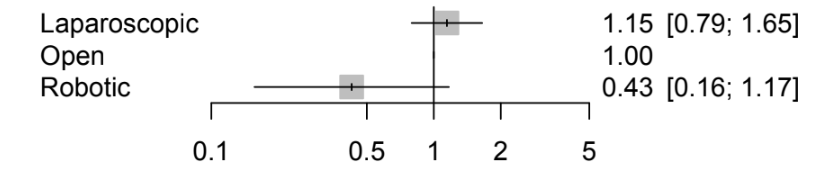** | 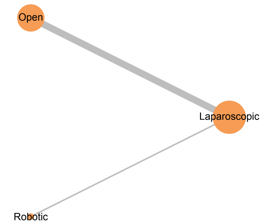 |
| **C** | 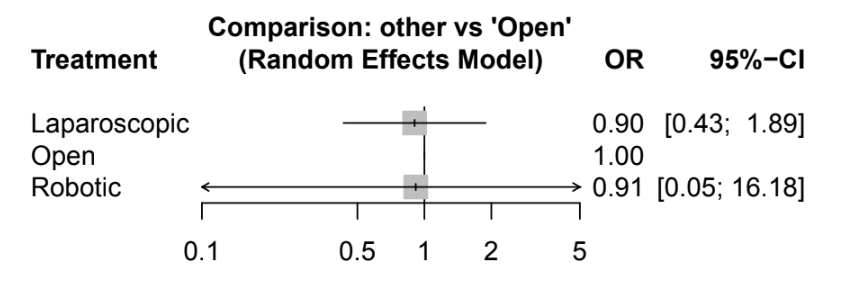 | 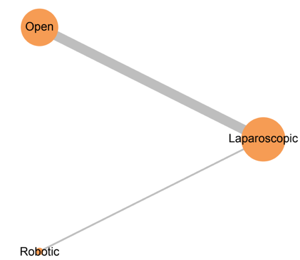 |
| **D** | **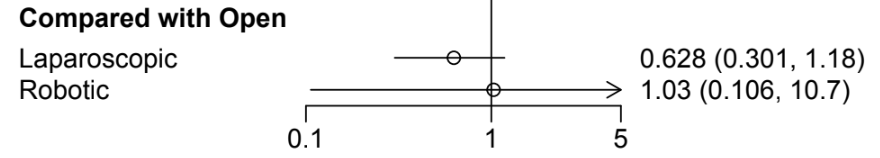** | 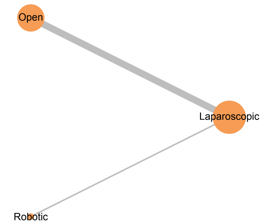 |
| **E** | **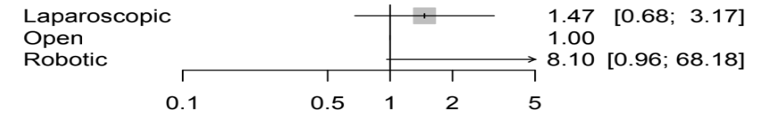** | 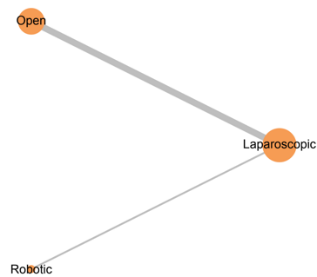 |
| **F** | **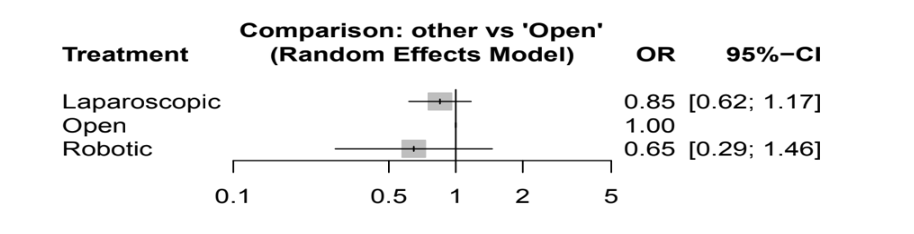** | 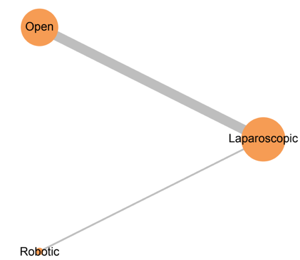 |
| **G** | **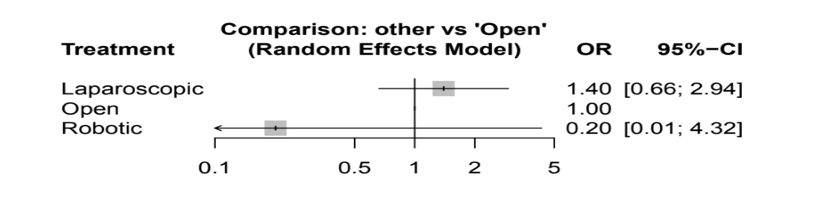** | 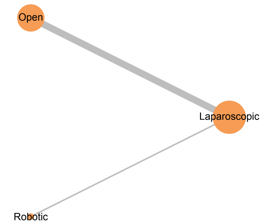 |
| **H** | N/A | 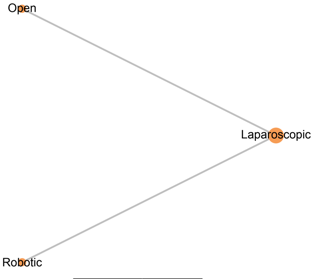 |
| **I** | **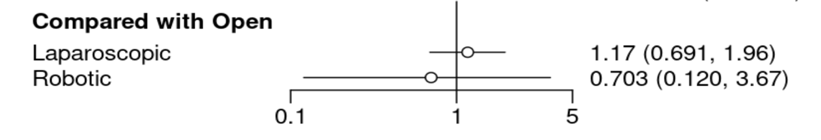** | 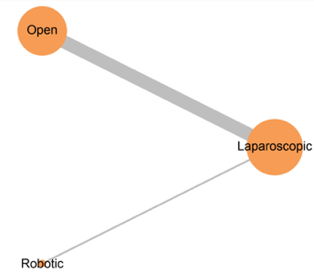 |
| **J** | **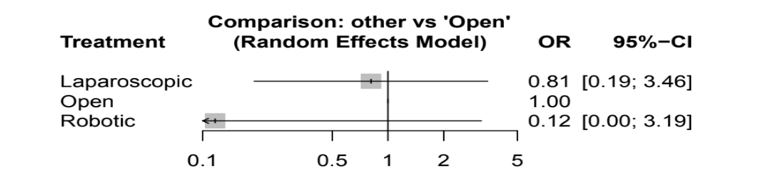** | **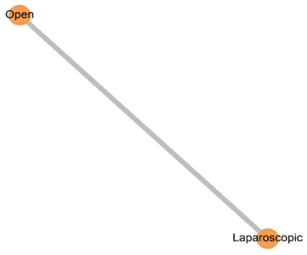** |

**Supplementary Appendix 5: Intraoperative data**

Iindividual studies with respect to Intraoperative data (A) Operative Time, (B) Intraoperative blood loss, (C) distance from the proximal margin, (D) distance from the distal margin and (E) Length of incision**.**

| **A** | **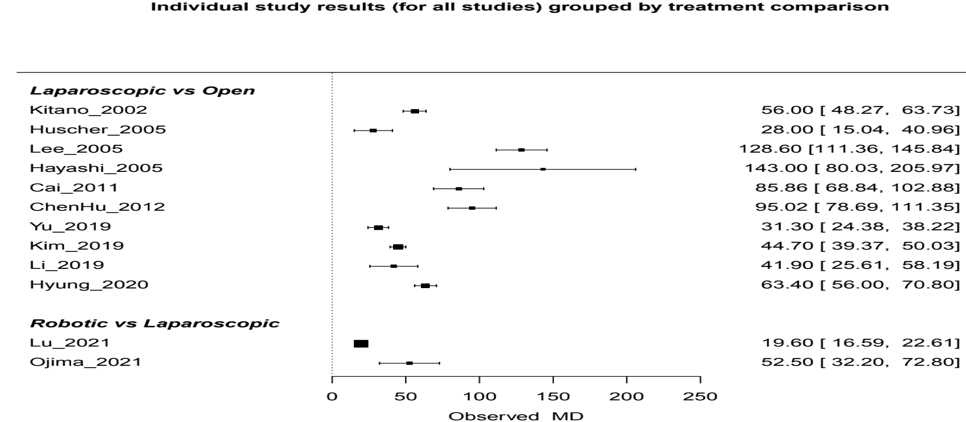** |
| --- | --- |
| **B** | **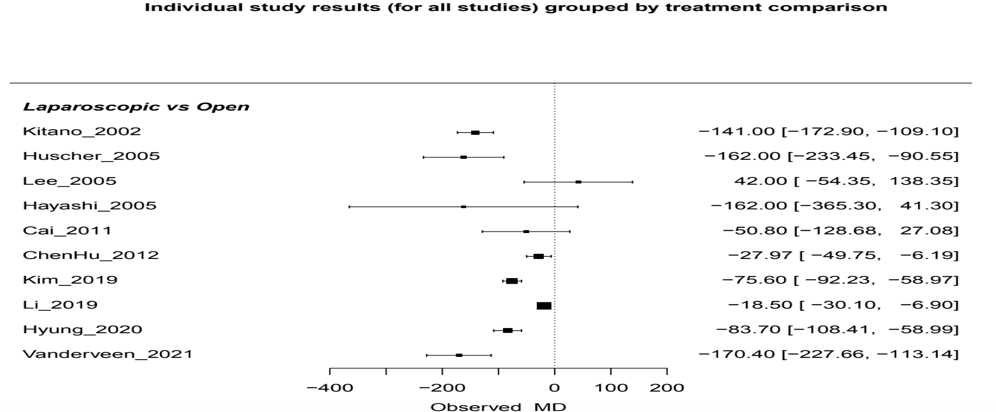** |
| **C** | **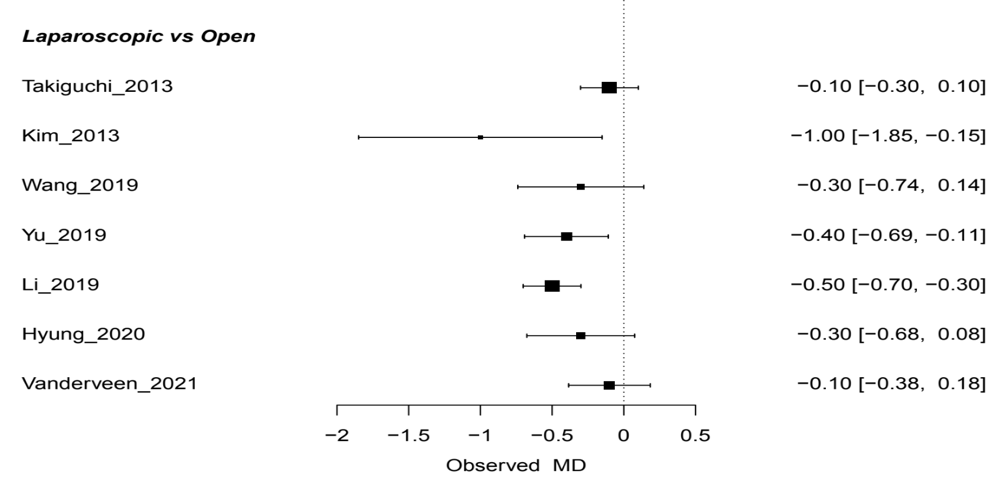** |
| **D** | **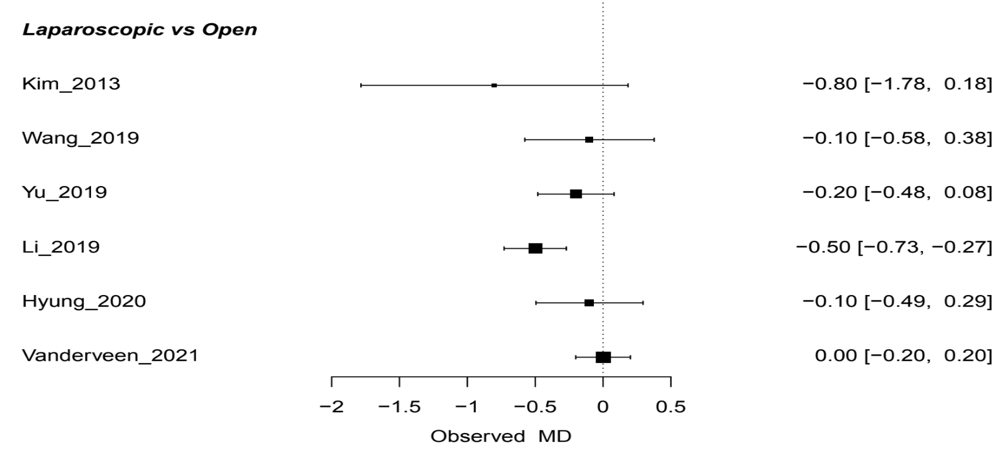** |
| **E** | **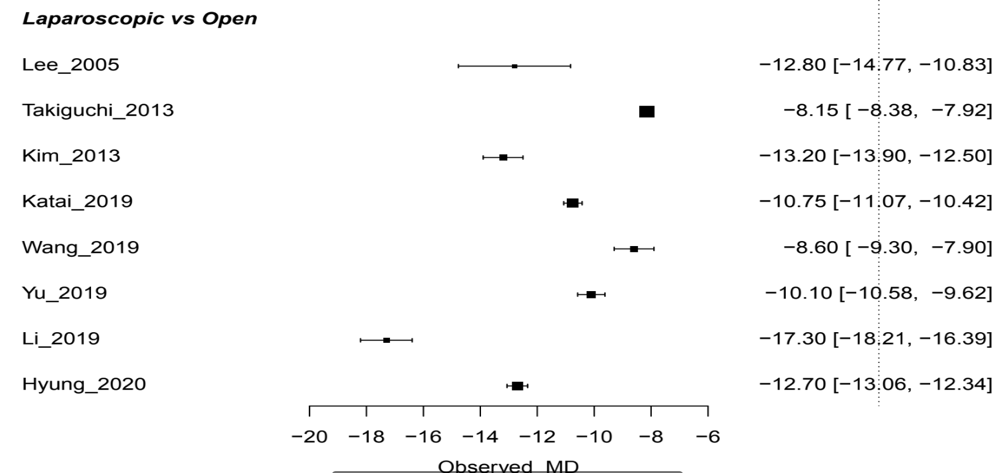** |

**Supplementary Appendix 6: Postoperative data**

Forest and network plots with respect to Recovery data (A) length of hospital stay, (B) days till sips, (C) days to solids., (D) days to first flatus, (E) days till first ambulation, (F) readmission.

| **Outcome** | **Forrest Plot** | **Network plot** |
| --- | --- | --- |
| **A** | 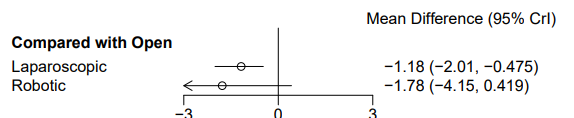 | **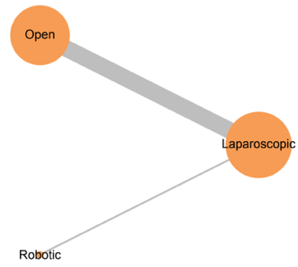** |
| **B** | **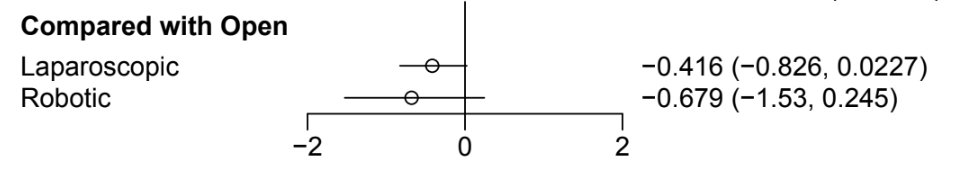** | **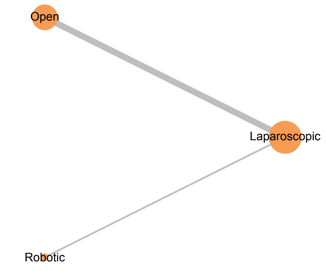** |
| **C** | 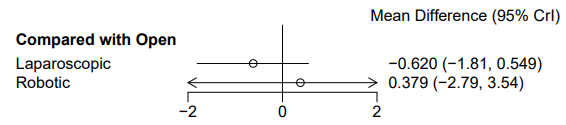 | **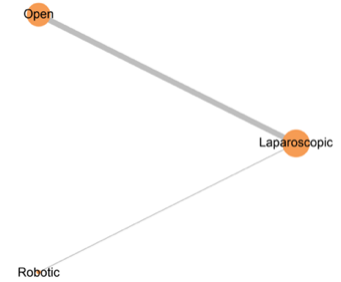** |
| **D** | 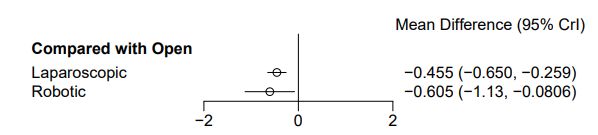 | **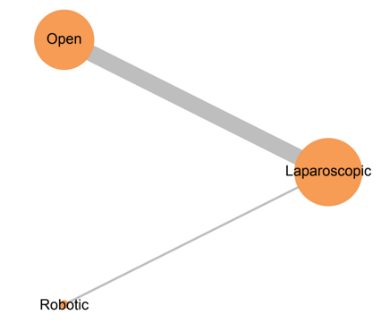** |
| **E** | 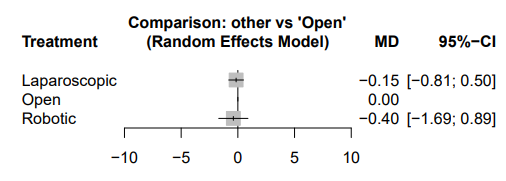 | **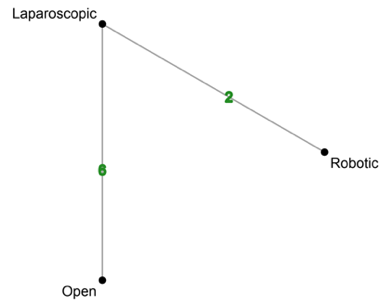** |
| **F** | **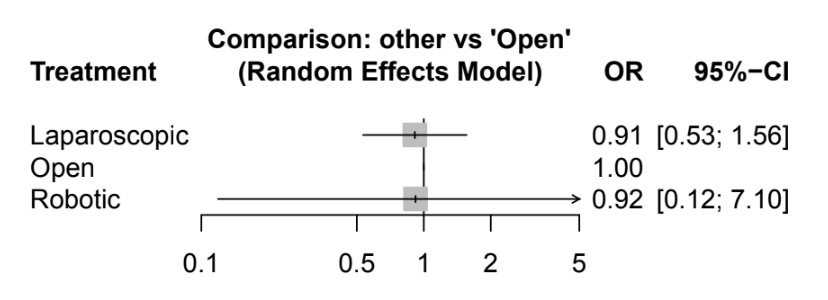** | **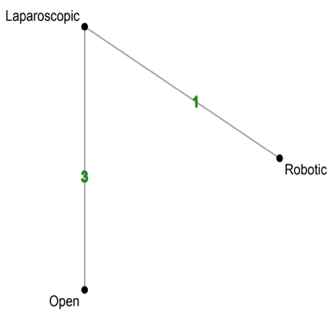** |

**Supplementary Appendix 7: Recovery measures data**

Network plots and individual studies with respect to **Recovery measures** (A) Length of stay, (B) Days till sips, (C) Days till solids, (D) Days till flatus (E) Days till ambulation and (F) Readmission

| **A** | **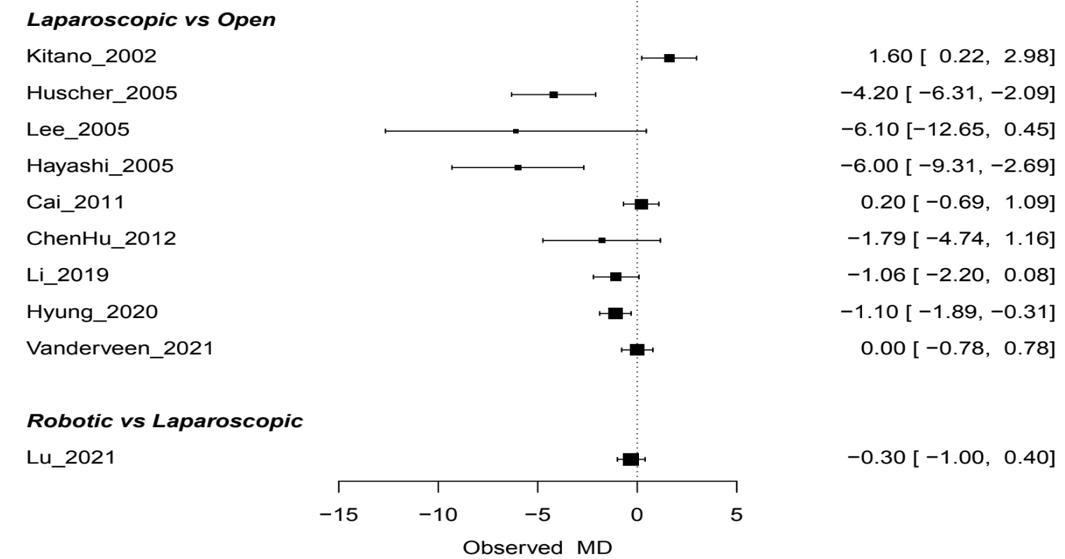** |
| --- | --- |
| **B** | **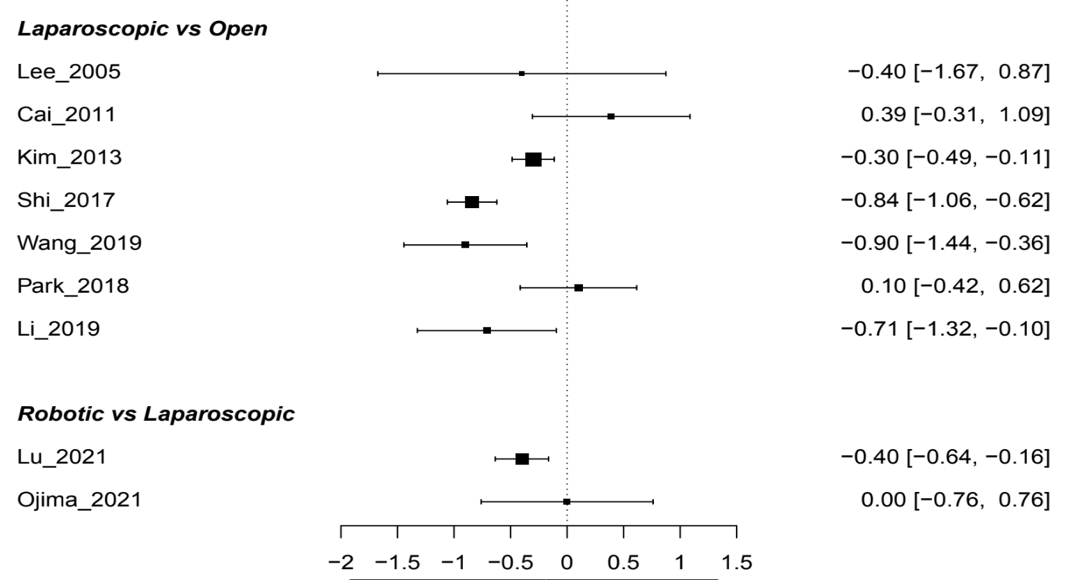** |
| **C** | N/A |
| **D** | **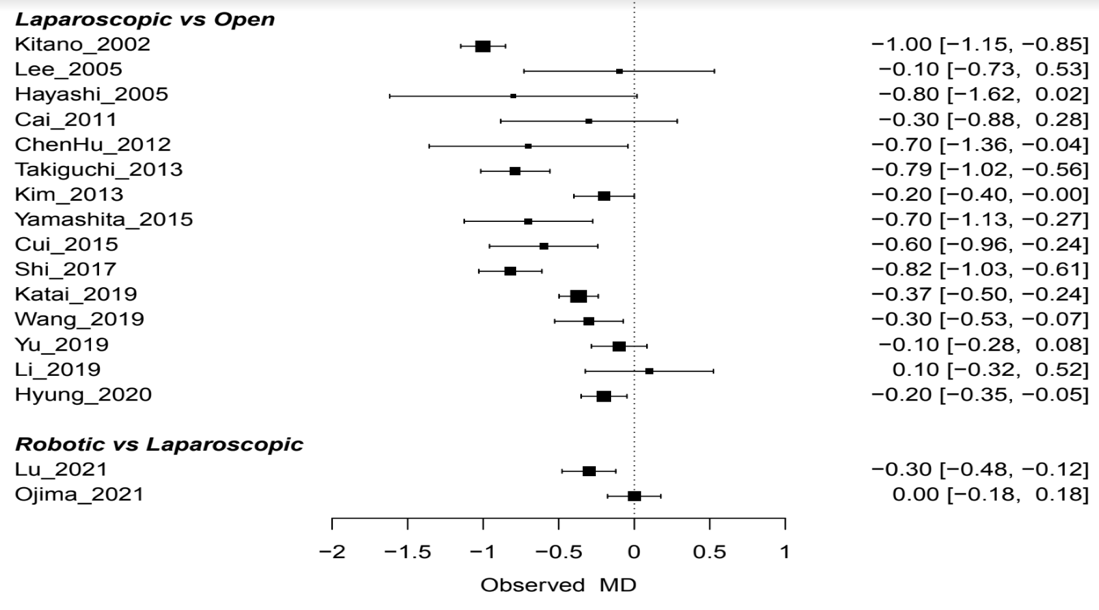** |
| **E** | **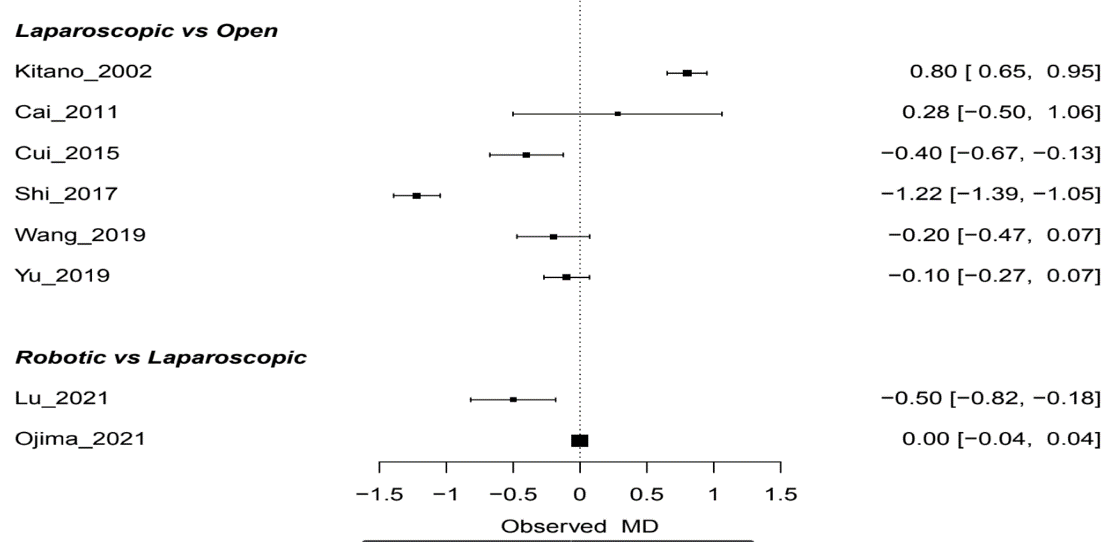** |
| **F** | **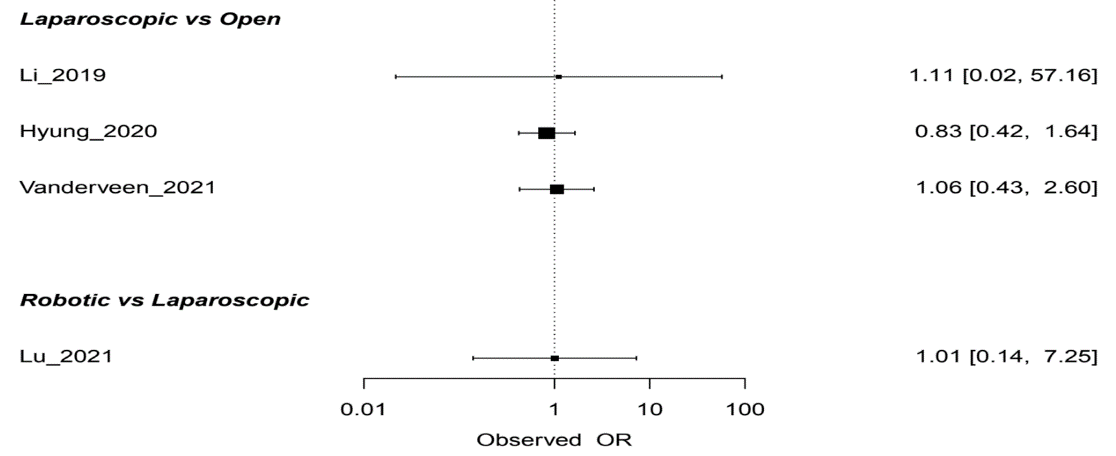** |

**Supplementary Appendix 8: Recovery measures data**

Forest and network plots with respect to Other outcome measure (A) Tumour size and (B) Cost

| **Outcome** | **Forrest Plot** | **Network plot** |
| --- | --- | --- |
| **A** | N/A | 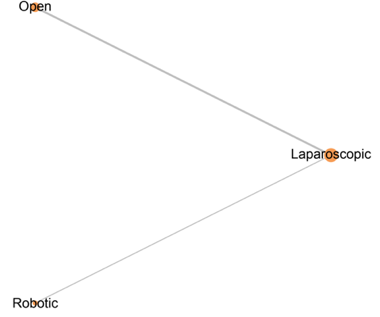 |
| **B** | N/a  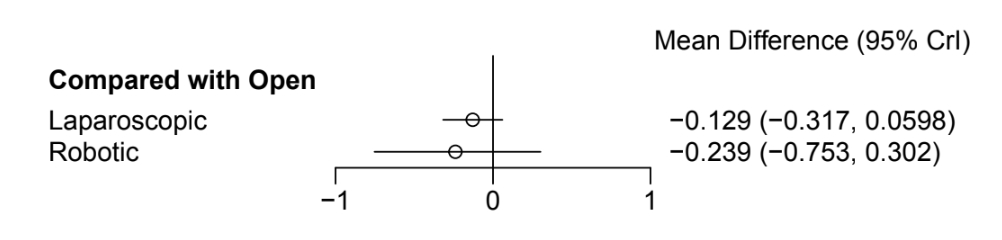 | 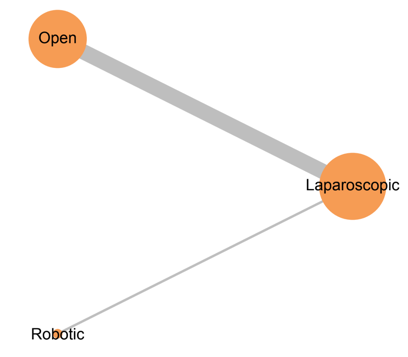 |

**Supplementary Appendix 9:**

Network plots and individual studies with respect to **other measures** (A) Cost (B) Tumour size

| A | 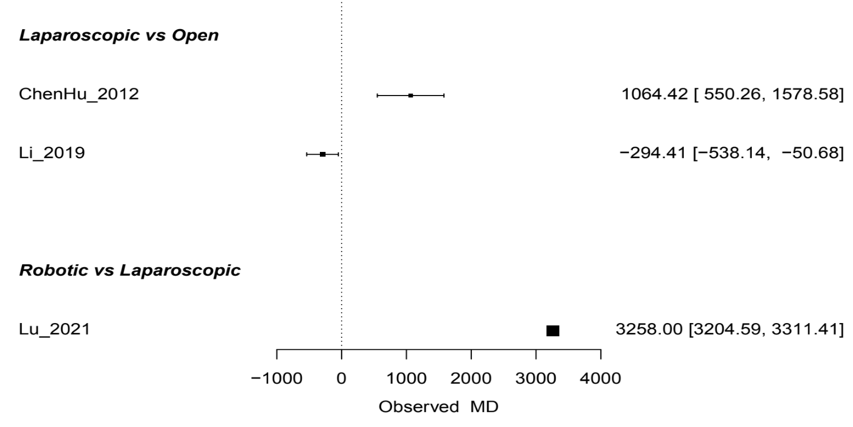 |
| --- | --- |
| B | N/A |

**Supplementary Appendix 10: Risk of Bias Assessment**

| **Author** | Random sequence  generation (SB) | Allocation  concealment  (SB) | Blinding of  participants and  personnel (PB) | Blinding of  outcome  assessment (DB) | Incomplete  outcome data  (AB) | Selective  reporting  (RB) | Other bias |
| --- | --- | --- | --- | --- | --- | --- | --- |
| **Kitano 2002** | unclear | + | unclear | unclear | + | + | + |
| **Huscher 2005** | unclear | unclear | unclear | unclear | + | + | + |
| **Lee 2005** | + | unclear | unclear | unclear | + | + | + |
| **Hayashi 2005** | unclear | + | - | unclear | + | + | + |
| **Cai 2011** | unclear | unclear | unclear | unclear | + | + | + |
| **Chen Hu 2012** | unclear | unclear | - | + | + | + | + |
| **Takiguchi 2013** | unclear | + | - | - | + | + | + |
| **Kim 2013** | + | + | - | unclear | + | + | + |
| **Syamashita 2015** | + | + | - | - | + | + | + |
| **Cui 2015** | + | + | unclear | unclear | - | + | + |
| **Aoyama 2014** | unclear | unclear | unclear | unclear | + | + | + |
| **Shi 2017** | + | + | - | - | + | + | + |
| **Katai 2019** | + | + | - | unclear | + | - | + |
| **Wang 2019** | unclear | unclear | - | - | unclear | - | unclear |
| **Park 2018** | + | + | - | unclear | + | - | + |
| **Yu 2019** | + | + | - | - | + | - | + |
| **Kim 2019** | + | + | - | - | - | - | + |
| **Li 2019** | + | + | unclear | + | + | _+ | + |
| **Hyung 2020** | + | + | - | + | + | + | + |
| **Vanderveen 2021** | + | + | - | unclear | + | - | + |
| **Jun Lu 2021** | + | + | + | - | + | + | + |
| **Ojima 2021** | + | + | unclear | + | + | + | + |

**References**

1. Shi Y, Xu X, Zhao Y, Qian F, Tang B, Hao Y, et al. Long-term oncologic outcomes of a randomized controlled trial comparing laparoscopic versus open gastrectomy with D2 lymph node dissection for advanced gastric cancer. Surgery. 2019;165(6):1211-6.

2. Kim YW, Yoon HM, Yun YH, Nam BH, Eom BW, Baik YH, et al. Long-term outcomes of laparoscopy-assisted distal gastrectomy for early gastric cancer: result of a randomized controlled trial (COACT 0301). Surg Endosc. 2013;27(11):4267-76.

3. Yamashita K, Sakuramoto S, Kikuchi S, Futawatari N, Katada N, Hosoda K, et al. Laparoscopic versus open distal gastrectomy for early gastric cancer in Japan: long-term clinical outcomes of a randomized clinical trial. Surg Today. 2016;46(6):741-9.

4. Hyung WJ, Yang HK, Park YK, Lee HJ, An JY, Kim W, et al. Long-Term Outcomes of Laparoscopic Distal Gastrectomy for Locally Advanced Gastric Cancer: The KLASS-02-RCT Randomized Clinical Trial. J Clin Oncol. 2020;38(28):3304-13.

5. Kim HH, Han SU, Kim MC, Kim W, Lee HJ, Ryu SW, et al. Effect of Laparoscopic Distal Gastrectomy vs Open Distal Gastrectomy on Long-term Survival Among Patients With Stage I Gastric Cancer: The KLASS-01 Randomized Clinical Trial. JAMA Oncol. 2019;5(4):506-13.

6. Katai H, Mizusawa J, Katayama H, Morita S, Yamada T, Bando E, et al. Survival outcomes after laparoscopy-assisted distal gastrectomy versus open distal gastrectomy with nodal dissection for clinical stage IA or IB gastric cancer (JCOG0912): a multicentre, non-inferiority, phase 3 randomised controlled trial. Lancet Gastroenterol Hepatol. 2020;5(2):142-51.

7. Yu J, Huang C, Sun Y, Su X, Cao H, Hu J, et al. Effect of Laparoscopic vs Open Distal Gastrectomy on 3-Year Disease-Free Survival in Patients With Locally Advanced Gastric Cancer: The CLASS-01 Randomized Clinical Trial. JAMA. 2019;321(20):1983-92.

8. Ojima T, Nakamura M, Hayata K, Kitadani J, Katsuda M, Takeuchi A, et al. Short-term Outcomes of Robotic Gastrectomy vs Laparoscopic Gastrectomy for Patients With Gastric Cancer: A Randomized Clinical Trial. JAMA Surg. 2021;156(10):954-63.

9. Kuo Yang, Lijie Lu, Huayi Liu, Xiujuan Wang, Ying Gao, Liu Yang, Yupeng Li, Meiling Su, Ming Jin & Samiullah Khan (2021) A comprehensive update on early gastric cancer: defining terms, etiology, and alarming risk factors, Expert Review of Gastroenterology & Hepatology, 15:3, 255-273, DOI: 10.1080/17474124.2021.1845140.

10. Stewart, Camille et al. “Multimodality management of locally advanced gastric cancer-the timing and extent of surgery.” Translational gastroenterology and hepatology vol. 4 42. 30 May. 2019, doi:10.21037/tgh.2019.05.02.

11. Washington, K. 7th Edition of the AJCC Cancer Staging Manual: Stomach. Ann Surg Oncol 17, 3077–3079 (2010). https://doi.org/10.1245/s10434-010-1362-z.

12. Armstrong EC. The well-built clinical question: the key to finding the best evidence efficiently. WMJ. 1999;98(2):25-28.

13. Dindo, Daniel MD; Demartines, Nicolas MD; Clavien, Pierre-Alain MD, PhD, FRCS, FACS Classification of Surgical Complications, Annals of Surgery: August 2004 - Volume 240 - Issue 2 - p 205-213 doi: 10.1097/01.sla.0000133083.54934.ae

14. Hummel R, Bausch D. Anastomotic Leakage after Upper Gastrointestinal Surgery: Surgical Treatment. Visc Med. 2017;33(3):207-11.

15. Lee SW, Tanigawa N, Nomura E, Tokuhara T, Kawai M, Yokoyama K, et al. Benefits of intracorporeal gastrointestinal anastomosis following laparoscopic distal gastrectomy. World J Surg Oncol. 2012;10:267.
